# Effect of manuka honey on biofilm-associated genes expression during methicillin-resistant *Staphylococcus aureus* biofilm formation

**DOI:** 10.1038/s41598-020-70666-y

**Published:** 2020-08-11

**Authors:** Barbara Kot, Hubert Sytykiewicz, Iwona Sprawka, Małgorzata Witeska

**Affiliations:** Institute of Biological Sciences, Faculty of Exact and Natural Sciences, Siedlce University of Natural Sciences and Humanities, 14 Bolesława Prusa Str., 08-110 Siedlce, Poland

**Keywords:** Microbiology, Molecular biology

## Abstract

Methicillin-resistant *Staphylococcus aureus* (MRSA) are among the most important biofilm-forming pathogens responsible for hard-to-treat infections. Looking for alternatives to antibiotics that prevent biofilm formation, we investigated the effects of manuka honey on the transcriptional profile of genes essential for staphylococcal biofilm formation using qRT-PCR. mRNA from two hospital MRSA strains (strong and weak biofilm producer) were isolated after 4, 8, 12 and 24 h from cells grown in biofilm. Manuka honey at 1/2 minimum biofilm inhibition concentration (MBIC) significantly reduced MRSA cell viability in biofilm. Manuka honey downregulated the genes encoding laminin- (*eno*), elastin- (*ebps*) and fibrinogen binding protein (*fib*), and *icaA* and *icaD* involved in biosynthesis of polysaccharide intercellular adhesin in both weakly and strongly adhering strain compared to the control (untreated biofilm). Expression levels of *cna* (collagen binding protein) and *map/eap* (extracellular adherence protein—Eap) were reduced in weakly adhering strain. The lowest expression of investigated genes was observed after 12 h of manuka honey treatment at 1/2 MBIC. This study showed that the previously unknown mechanism of manuka honey action involved inhibition of *S. aureus* adhesion due to reduction in expression of crucial genes associated with staphylococcal biofilm.

## Introduction

Chronic wound infections are difficult to treat and therefore pose a serious burden to society and the healthcare system. In the United States alone, such wounds occur in 6.5 million people (their frequency significantly increases), and their treatment costs over US $25 billion yearly^1^. In chronic wound infections, bacterial cells predominantly exist as biofilm in which they can tolerate up to 10–1,000 times higher concentrations of antimicrobials than planktonic cells, which makes them difficult to eradicate using antibiotic therapy^2^. Bacteria in biofilm, enclosed in a self-produced exopolysaccharide glycocalyx, usually show no sensitivity to antibiotic treatment. Antimicrobial resistance of biofilms is related to reduced antibiotic penetration into multiple layers of bacteria in biofilm, different growth rates of bacterial cells, nutrient gradients within the biofilm^2^ and the presence of dormant variants (persister phenomenon) highly tolerant to antibiotics^3^. Methicillin-resistant *Staphylococcus aureus* (MRSA) are responsible for hard-to-treat infections including chronic wound infections. The prevalence of MRSA colonization or infection in chronic ulcers varies between 7 and 30%^4^. Research conducted by Pereira-Franchi et al.^5^ in Brazil showed that in the chronic wounds, prevalence rates for *S. aureus* was 51.5% and for MRSA 8.7%. While, the study concerning determination of microbiological profile of diabetic foot ulcers, showed that MRSA was present in 23% of wounds^6^.

MRSA show resistance to β-lactam antibiotics but also may be resistant to other antibiotic groups, such as aminoglycosides, fluoroquinolones, macrolides, tetracycline and chloramphenicol^7^. The prevalence of multidrug-resistant MRSA strains and ability of MRSA to form biofilm which is involved in all chronic infections make MRSA a serious threat to human health. On the other hand, limited development of antimicrobial agents in recent times increased the necessity to search for alternative remedies to complement or replace antibiotics.

Manuka honey derived from New Zealand flowers of *Leptospermum scoparium* has been known for its bactericidal activity^8,9^. Antibacterial effects of manuka honey are considered to be related to substantial content of the reactive dicarbonyl methylglyoxal (MGO) and other antimicrobial compounds including bee defensin-1, various phenolic compounds and complex carbohydrates. Additional antimicrobial factors such as osmotic stress resulting from the high sugar concentration, low protein content, low pH, the presence of hydrogen peroxide (H_2_O_2_) produced by the bee-derived enzyme glucose oxidase are also considered as contributing to manuka honey antibacterial activity^9–11^. In the literature a few studies are available concerning the effects of manuka honey on biofilm development by various strains of *S. aureus* of different biofilm-forming ability^12,13^. The activity of key antibacterial components of manuka honey against different bacteria was also investigated^10,14,15^ but changes in MRSA gene expression in the presence of manuka honey, according to our knowledge, were described in only one study^16^.

In our earlier research, we showed that the expression of specific staphylococcal biofilm-associated genes was significantly higher in biofilm than in planktonic conditions^17^. Therefore, looking for alternatives to antibiotics that prevent biofilm formation, in the present study we focused on investigation of the effects of manuka honey on the transcriptional profile of genes that are essential for staphylococcal biofilm formation. Among the genes encoding microbial surface components recognizing adhesive matrix molecules (MSCRAMMs) we investigated the expression of *eno*, *ebps*, *fib* and *cna* genes in the presence of manuka honey. Moreover, the effects of manuka honey on expression of *map/eap* gene encoding extracellular adherence protein (Eap)—an anchorless protein belonging to the group of SERAMs (secretable expanded repertoire adhesive molecules) and on expression of genes from the *ica* operon (*icaA* and *icaD*) in different time points during biofilm formation, were also investigated.

α-enolase, encoded by the *eno* gene, allows *S. aureus* to bind to laminin, a major component of the basal membrane of the blood vessels. Adherence to the blood vessel walls allows the dissemination of staphylococcal cells by blood and may contribute to host tissue invasion. Besides, α-enolase also acts as a plasminogen receptor. Plasminogen activation may result in laminin degradation in restricted areas^18^. The elastin binding protein of *S. aureus* (EbpS) is encoded by the *ebps* gene. Binding of *S. aureus* to elastin which is a major component of the elastic fiber of extracellular matrix, promotes colonization of mammalian tissues by this microorganism^19^. The *fib* gene encodes the fibrinogen binding protein (Fib)^20^. Fibrinogen is a blood glycoprotein that mediates platelet adherence, aggregation and clotting in sites of injury. Adhesion of *S. aureus* to fibrinogen is an important factor promoting wound infection, endocarditis and also foreign body infection because fibrinogen is one of the main proteins deposited on implanted biomaterials^21^. Collagen Cn-binding protein (Cna) plays an important role in staphylococcal pathogenesis, both as an adherence factor and as an immune evasion factor^22^. Cna is a cell-wall-anchored protein participating in the adhesion of *S. aureus* to collagen-rich tissues^23^. Collagen provides the structural support for tissues and serves as a scaffold for the assembly of extracellular matrices. According to Kang et al.^24^ Cna binds to complement protein C1q and inhibits the activation of the complement system.

Map^25^ or Eap^26^ is a staphylococcal surface protein of different molecular mass (either 72 or 60 kDa) in different strains^27^. Sequence analysis showed high homology of Map and Eap, and Hussain et al.^28^ stated that they are analogues of the same protein. Map is associated but not covalently linked to the *S. aureus* cell surface^27^ and can interact with various glycoproteins of extracellular matrix (ECM), such as fibronectin, fibrinogen, sialoprotein and some collagens^29^. Hussain et al.^30^ reported that Map is also involved in adhesion of *S. aureus* to fibroblasts as well as in bacteria internalization.

In the accumulative phase of biofilm formation, intercellular adhesive mechanisms are expressed. In this phase, most staphylococcal cells have no direct contact with the surface and remain in the biofilm structure due to the main adhesin, the polysaccharide intercellular adhesin (PIA) encoded by *ica* operon. PIA is composed of β-1,6-linked *N*-acetylglucosamine residues and an anionic fraction with lower content of non-*N*-acetylated d-glucosaminyl residues. PIA promotes adhesive interactions among bacterial cells^31,32^. Gene *icaA* from *ica* operon encodes low activity N-acetylglucosaminyl-transferase. Co-expression of the *icaA* gene and *icaD* significantly increases the activity of this enzyme and slime production^33^.

## Methods

### Bacterial strains

Two MRSA strains were obtained directly from hospital in Siedlce (Poland) and patients were not involved in the present study. The strains were isolated from wound and anus in 2017. The genes: *mecA* responsible for resistance against β-lactam antibiotics, *eno*, *ebps*, *fib* encoding MSCRAMMs, and genes from *ica* operon (*icaA* and *icaD*) were identified according to methods described earlier^17^. The primers specific for the *cna* gene encoding collagen binding protein and *map/eap* gene encoding the extracellular adhesion protein synthesized at DNA-Gdańsk (Gdańsk, Poland), are listed in Table 1. PCR conditions for *cna* and *map/eap* were the same as for genes described by Kot et al.^17^ The strain 27,887 isolated from wound formed strong biofilm on polystyrene, while the strain 1,037 isolated from anus weakly adhered to polystyrene^17^.

**Table 1:** Oligonucleotide primers used in PCR.

Primers	Sequence (5′ → 3′)	Amplicon size (bp)	References
*cna* (F)	GTCAAGCAGTTATTAACACCAGAC	423	Tristan et al.^20^
*cna* (R)	AATCAGTAATTGCACTTTGTCCACTG		
*map/eap* (F)	TAACATTTAATAAGAATCAA	943–9	Rohde et al.^52^
*map/eap*(R)	CCATTTACTGCAATTGT		

### Determination of minimum inhibitory concentrations (MIC) and minimum bactericidal concentrations (MBC) of manuka honey

Certified manuka honey Mn550 (the number corresponds to minimal methylglyoxal (MGO) content in mg/kg) was obtained from Propharma (Warsaw, Poland) and was stored at room temperature in the dark. The solutions of honey were prepared weight per volume (w/v) in Mueller–Hinton Broth (MHB; BBL, Becton Dickinson, Sparks, Md., Franklin Lakes, NJ, USA) and were used in antibacterial activity assay within 30 min from preparation. Serial twofold dilutions of manuka honey were prepared to obtain concentrations ranging from 50 to 0.8%. Determination of MIC and MBC of manuka honey was carried out according to method described by Kot et al.^34^.

### Determination of minimum biofilm inhibition concentrations (MBIC) and minimum biofilm eradication concentrations (MBEC) of manuka honey

Manuka honey was diluted in Tryptic-Soy Broth (TSB; BBL, Becton Dickinson, Sparks, Md.) with 0.5% glucose. Serial twofold dilutions of honey were prepared to obtain concentrations ranging from 50% to 1.56%. Determination of MBIC and MBEC of manuka honey was carried out according to method described earlier by Kot et al.^35^.

### Manuka honey inhibiting biofilm formation assay in different time intervals

The effectiveness of manuka honey against biofilm formation was evaluated in tissue culture polystyrene 96-well plates (Nunclon, Roskilde, Denmark). Suspensions of staphylococcal cells in sterile phosphate-buffered saline (PBS, pH 7.4) were diluted in order to prepare suspensions containing about 10^8^ CFU/mL in TSB with 0.5% glucose and manuka honey at final concentrations of 1/4 MBIC and 1/2 MBIC. Subsequently, bacterial cell suspensions (200 µL) were transferred in five replicates to wells of plate. After that, the plates were incubated at 37 °C for 4, 8, 12 and 24 h statically. Control of bacterial growth was performed in the wells with bacterial cell suspensions without manuka honey, as well as control of sterility of the media (wells without addition of bacterial cell suspension). Bacterial cell viability in biofilms, after the appropriate incubation period, was determined by the resazurin microtiter-plate assay as described earlier^35^. Metabolic assay is excellent for the quantification of bacterial viability in biofilm, and the amount of metabolite produced by a biofilm depends on both the metabolic activity of the individual bacteria and the number of live bacteria in the biofilm. The resazurin assay is based on the reduction of resazurin, a blue dye that can be reduced by metabolically active cells to pink resorufin, which is fluorescent. For this purpose, after the incubation period, the medium was removed, and non-adherent bacterial cells were discarded by washing the biofilms twice with 250 µL of sterile PBS. Subsequently, TSB with 0.5% glucose (190 µL) and 10 µL of resazurin (Sigma-Aldrich, Steinheim, Germany) aquatic solution (0.01%) were added to each well with biofilm. After 2 h incubation of the microplates with resazurin in darkness at 37 °C, absorbance was measured at 492 nm in microplate reader (Apollo LB913, Berthold Technologies, Bad Wildbad, Germany). Each assay was performed three times and the results were averaged. The influence of manuka honey on the bacterial viability in biofilm was evaluated by comparison of the metabolic activity of biofilm with manuka honey to the metabolic activity of biofilm that was not exposed to manuka honey. The results are presented as percentages of biofilm metabolic activity reduction, according to Borges et al.^36^.

### Growth conditions of MRSA strains

MRSA strains were grown in TSB with 0.5% glucose at 37 °C for 18 h, and after that, the cultures were inoculated on TSA with 0.5% glucose and incubated at 37 °C for the next 18 h. Subsequently, bacterial cells of each strain were suspended in the PBS (pH 7.4) to get the optical density OD565 = 3.2 (densitometer DEN-1, Biosan, Riga, Latvia). The obtained suspensions were diluted with TSB containing 0.5% glucose and manuka honey at final concentrations of 1/4 MBIC and 1/2 MBIC in order to prepare cell suspensions containing about 10^8^ CFU/mL. Aliquots of 1 mL bacterial cell suspensions with manuka honey were transferred in eight replicates to wells of a tissue culture polystyrene 24-well plate (Nunclon) and incubated at 37 °C. Control of bacterial growth was performed in the wells with bacterial cell suspensions in TSB with 0.5% glucose.

### Preparation of the lysate of bacterial cells, RNA extraction and cDNA synthesis

Bacterial cells growing in biofilm in the presence or without manuka honey were harvested at four different times (4, 8, 12 and 24 h). The bacterial cells were rinsed with sterile PCR-grade water, scraped with pipette and suspended in appropriate volume of RNAprotect Bacteria Reagent (QIAGEN, Hilden, Germany) in order to prepare bacterial suspensions containing about 5 × 10^8^ CFU/ml. Preparation of the lysate of bacterial cells, RNA extraction and cDNA synthesis were performed according to method described earlier by Kot et al.^17^.

### Gene expression quantification

Expression level of *icaA*, *icaD*, *eno*, *ebps*, *fib*, *can* and *map/eap* genes of MRSA strains was performed using real-time qRT-PCR technique according to the protocol presented earlier by Kot et al.^17^ The list of primers and TaqMan fluorescent probes that were used in the study is presented in Table 2. The *rpoB* gene, encoding RNA polymerase subunit, was used as the internal reference.

**Table 2:** Sequences of primers designed for real-time qRT-PCR analyses.

Genes	Accession no. (GenBank)	Sequences of primers and probes
*icaA*	SAB2541 (K11936)	F: CAATACTATTTCGGGTGTCTTCACTCT R: CAAGAAACTGCAATATCTTCGGTAATCAT P: 5′-FAM-CCCAGTAGCCAACATC-NFQ-3′
*icaD*	SAB2542 (K21461)	F: TCAAGCCCAGACAGAGGGAATA R: ACACGATATAGCGATAAGTGCTGTTT P: 5′-FAM-CCCAACGCTAAAATC-NFQ-3′
*eno*	AF065394.1	F: AAACTGCCGTAGGTGACGAA R: TGTTTCAACAGCATCTTCAGTACCTT P: 5′-FAM- TTCGCTCCTAAATTTG-NFQ-3′
*ebps*	SAB1343c	F: ACATTCAAATGACGCTCAAAACAAAAGT R: CTTATCTTGAGACGCTTTATCCTCAGT P: 5′-FAM- CAAGGCGAATAACTCG-NFQ-3′
*fib*	SAB1021 (K14200)	F: GAATATGGTGCACGTCCACAATT R: AAGATTTTGAGCTTGAATCAATTTTTGTTCTTTTT P: 5′-FAM-TCGCTGCTGGTTTATT-NFQ-3′
*cna*	M81736	F: GACTTACCGAAGTATGATGAAGGAAAGA R: ACCGTTGATGTCTGTTGTGTAGTC P: 5′-FAM-ACAGTGACCGAAGATC-NFQ-3′
*map/eap*	SAB1873c	F: GGAACGAAGAAAGTTATTGATTTGAAAGCA R: TTTTTGTATCTACGTTAATATCGATACTTTTAATATCACTTGA P: 5′-FAM-TCGCTGTGTAAATACC-NFQ-3′
*rpoB*	KY086792.1	F: CAGCTGACGAAGAAGATAGCTATGT R: ACTTCATCATCCATGAAACGACCAT P: 5′-TAGCACAAGCAAACTC-NFQ-3′

### Statistical analysis

All data were expressed as the mean (± SD). The obtained results were analysed using STATISTICA 12 software (StatSoft, Poland). Three-factorial analysis of variance (ANOVA) with consecutive Tukey’s test were applied to evaluate the significance of the effects of tested variables (strain, treatment, growth period) and their interactions on relative expression level of seven target genes (*icaA*, *icaD*, *eno*, *ebps*, *fib*, *can*, *map/eap*) of MRSA strains. Three-factorial analysis of variance (ANOVA) with consecutive Tukey’s post-hoc test were also used to evaluate the significance of effects of tested variables (strain, treatment, growth period) and their interactions on the metabolic activity of MRSA strains.

## Results

### Antibacterial activity of manuka honey against MRSA strains

The MRSA strains of different abilities to form biofilm on polystyrene showed similar susceptibility to manuka honey. MRSA strain (27,887) isolated from wound produced strong biofilm, while the strain (1,037) from anus was a weak biofilm producer. The values of MIC (minimum inhibitory concentrations), MBC (minimum bactericidal concentrations) and MBIC (minimum biofilm inhibition concentrations) for both strains were 12.5%. Higher concentration of manuka honey was required for total eradication of the biofilm and the MBEC value (minimum biofilm eradication concentration) was 25% for both strains (Table 3).

**Table 3:** Antibacteriassl activity of manuka honey against methicillin-resistant *Staphylococcus aureus* (MRSA) strains in planktonic culture and biofilm conditions.

Strain	Source	Biofilm	Manuka honey (%, w/v)			
			Planktonic cells		Biofilm	
			MIC	MBC	MBIC	MBEC
27,887	Wound	Strong	12.5	12.5	12.5	25.0
1,037	Anus	Weak	12.5	12.5	12.5	25.0

### The influence of manuka honey on bacterial cell viability in biofilm in different time intervals

**Figure 1:**
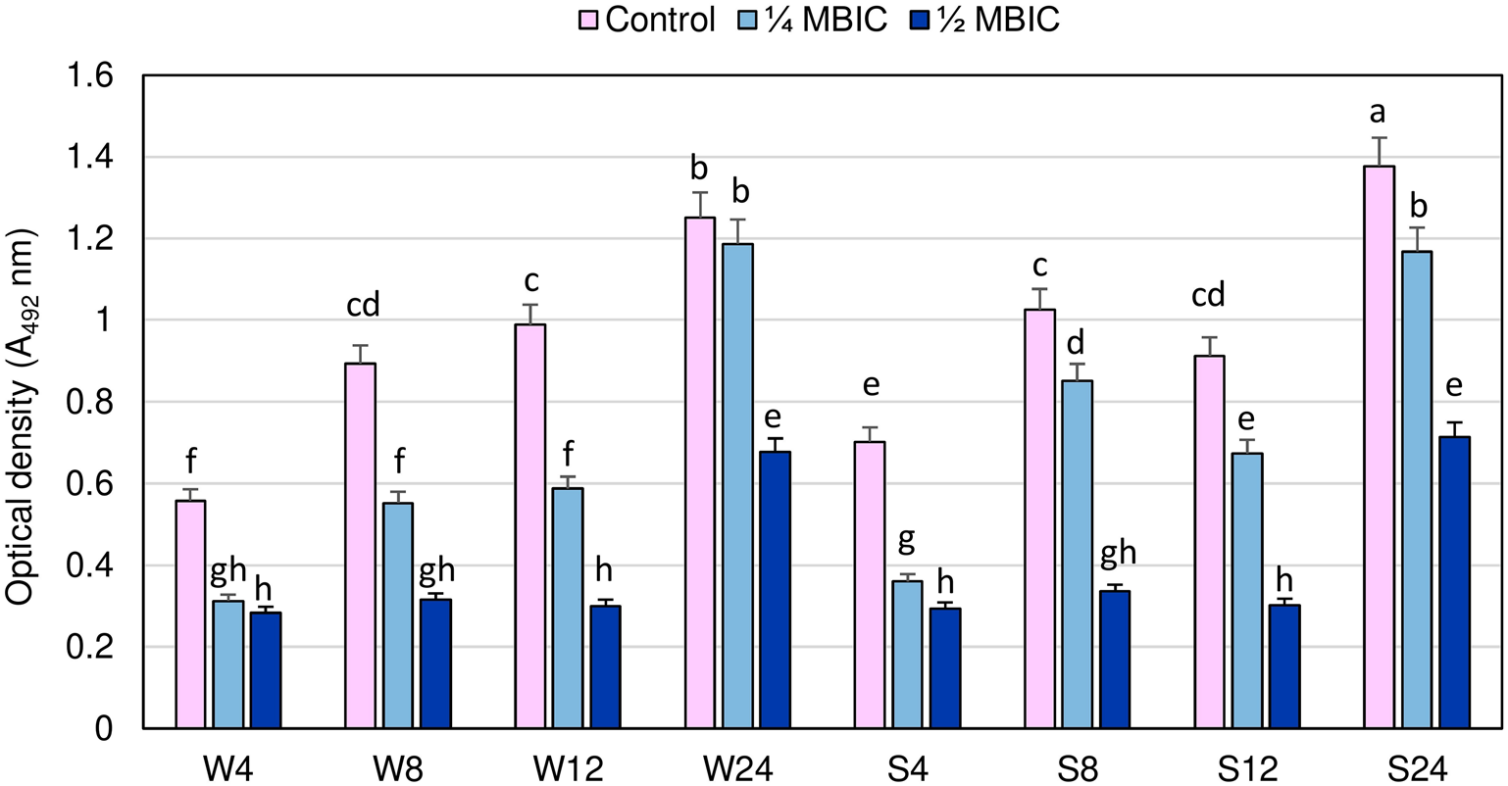
Bacterial cell viability in biofilms formed by MRSA strains in the presence of manuka honey at concentrations 1/4 and 1/2 minimum biofilm inhibition concentration (MBIC) in different time intervals. Different letters (a, b, c, d, e, f, g, h, cd, gh) denote significant differences in metabolic activity among investigated MRSA samples (Tukey’s test; *p* < 0.05). W: weak producer of biofilm; S: strong producer of biofilm; 4, 8, 12, 24: time of bacterial growth (h), MBIC: minimum biofilm inhibition concentration. Arithmetic mean and standard deviation, n = 3.

The viability of staphylococcal cells in the biofilm after manuka honey treatments at concentrations 1/4 and 1/2 MBIC in four time points is shown in Fig. 1. To evaluate the susceptibility of bacteria in biofilm to antimicrobials, it is essential to determine the amount of viable bacteria in the biofilm. We used metabolic assay appropriate to quantify bacterial viability in biofilm structure, in which the amount of metabolite produced by the biofilm depends on both metabolic activity of the individual bacteria and the number of live bacteria in the biofilm. Reduction of metabolic activity of the biofilm formed by a weakly adhering MRSA strain after 4, 8 and 12 h of manuka honey treatment with 1/4 MBIC compared to the control values at the same time were 44.06% ± 0.95%, 38.08% ± 4.07% and 40.48 ± 2.97%, respectively, and metabolic activity itself was significantly lower than in untreated biofilm. After 24 h manuka honey at 1/4 MBIC reduced metabolic activity of biofilm formed by this strain only in 4.89% ± 3.39% and the difference compared to untreated biofilm was insignificant (Fig. 1).

In strongly adhering MRSA strain, metabolic activity of biofilm treated with manuka honey at 1/4 MBIC in all investigated time points was significantly lower than in the control. However, the degree of metabolic activity reduction in biofilm formed by this strain after 8 and 12 h (20.46% ± 2.68% and 27.56% ± 2.55%, respectively) was lower than in the weakly adhering strain. Reduction of metabolic activity of both strains in the presence of manuka honey at 1/2 MBIC concentration did not significantly differ at the same time. Reduction of metabolic activity of these strains was significantly higher at 1/2 MBIC than at 1/4 MBIC. Maximum reduction of metabolic activity in both strains compared to the control values was observed after 8 and 12 h. After 8 and 12 h of manuka honey treatment at 1/2 MBIC metabolic activity of biofilm formed by the weakly adhering MRSA strain was reduced by 64.58% ± 0.8% and 69.5% ± 1.4%, respectively, while in the strongly adhering strain 67.2% ± 1.0% and 66.7% ± 1.0%, respectively. Significant increase in metabolic activity of both strains was observed after 24 h, although it was significantly lower than in untreated biofilm. Reduction in metabolic activity of the biofilm formed by the strongly adhering MRSA strain after 24 h manuka honey treatment at 1/2 MBIC compared to the control was 48.0% ± 3.2%, and in weakly adhering strain 45.8% ± 1.8%.

### Expression levels of genes associated with biofilm formation in the presence of manuka honey quantified by real-time qRT-PCR

**Figure 2:**
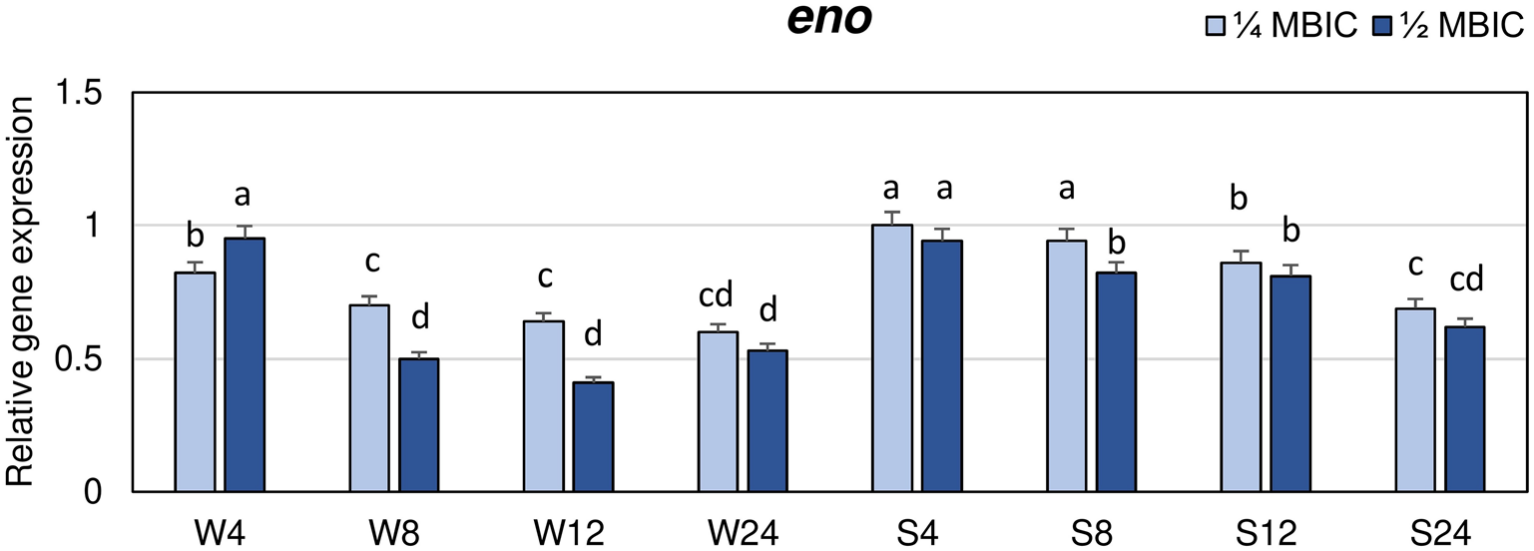
Transcriptional reprogramming of encoding laminin binding protein (*eno*) gene in biofilms of MRSA treated with manuka honey. Gene expression data were normalized to the *rpoB* reference gene. Results are shown as n-fold changes in the target gene expression compared to the control (untreated biofilm). Significant differences in relative gene expression levels among examined MRSA samples were marked by different letters (a, b, c, d, cd) (Tukey’s test; *p* < 0.05). W: weak producer of biofilm; S: strong producer of biofilm; 4, 8, 12, 24: time of bacterial growth (h); MBIC: minimum biofilm inhibition concentration. Arithmetic mean and standard deviation, n = 3.

The influence of manuka honey on expression levels of four MSCRAMM genes (*eno*, *ebps*, *fib* and *cna*), two genes from *ica* operon (*icaA*, *icaD*) and *map/eap* gene encoding extracellular adherence protein belonging to the group of SERAMs was investigated. The expression levels of these genes at 1/4 and 1/2 MBIC of manuka honey were measured after 4, 8, 12 and 24 h of biofilm growth, and they were compared to the control. The results are presented as the n-fold change of gene expression levels in biofilm treated with manuka honey in relation to expression levels in nonexposed biofilm. Manuka honey at 1/2 MBIC reduced the expression levels of the *eno* gene in biofilms formed by both strains (Fig. 2). In case of the weak biofilm producer, the highest decrease in *eno* expression was observed after 12 h (0.4 of the expression level in the control). Whereas, expression of the *eno* gene after 8 and 24 h at the presence of 1/2 MBIC was twice lower than in untreated biofilm. The values observed after 8, 12 and 24 h did not significantly differ. In the strongly adhering strain, expression level of *eno* gene at 1/2 MBIC of manuka honey was reduced less than in weakly adhering strain, and the lowest value was obtained after 24 h (0.6 of the control). Manuka honey at 1/4 MBIC also reduced the expression levels of the *eno* gene but after 8 and 12 h (weakly adhering strain) and after 8 h (strongly adhering strain) expression was significantly higher than at 1/2 MBIC. The lowest expression of *eno* in biofilm of strongly adhering strain at 1/4 MBIC was also observed after 24 h, similarly as at 1/2 MBIC.

**Figure 3:**
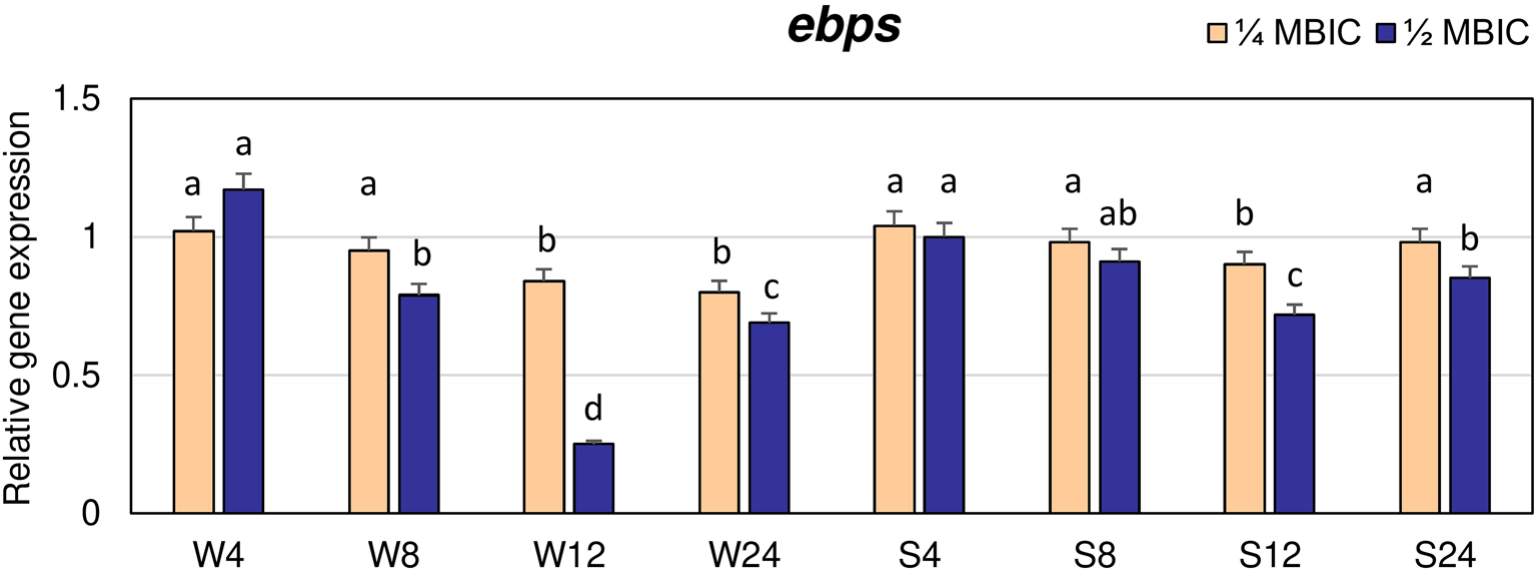
Transcriptional reprogramming of encoding elastin binding protein (*ebps*) gene in biofilms of MRSA treated with manuka honey. Gene expression data were normalized to the *rpoB* reference gene. Results are shown as n-fold changes in the target gene expression compared to the control (untreated biofilm). Significant differences in relative gene expression levels among examined MRSA samples were marked by different letters (a, b, c, d, ab) (Tukey’s test; *p* < 0.05). W: weak producer of biofilm; S: strong producer of biofilm; 4, 8, 12, 24: time of bacterial growth (h); MBIC: minimum biofilm inhibition concentration. Arithmetic mean and standard deviation, n = 3.

The highest decrease in expression of *ebps* gene by manuka honey at 1/2 MBIC was observed after 12 h. In the weakly adhering strain, at this time the *ebps* expression level was fourfold lower than in untreated biofilm, while in strong biofilm producer, the value was equal to 0.7 of expression level in the control (Fig. 3). The expression levels of *ebps* gene in both strains after 8 and 24 h were also lower compared to the control but in weakly adhering strain expression after 24 h was significantly lower at 1/4 and 1/2 MBIC than in strongly adhering strain.

**Figure 4:**
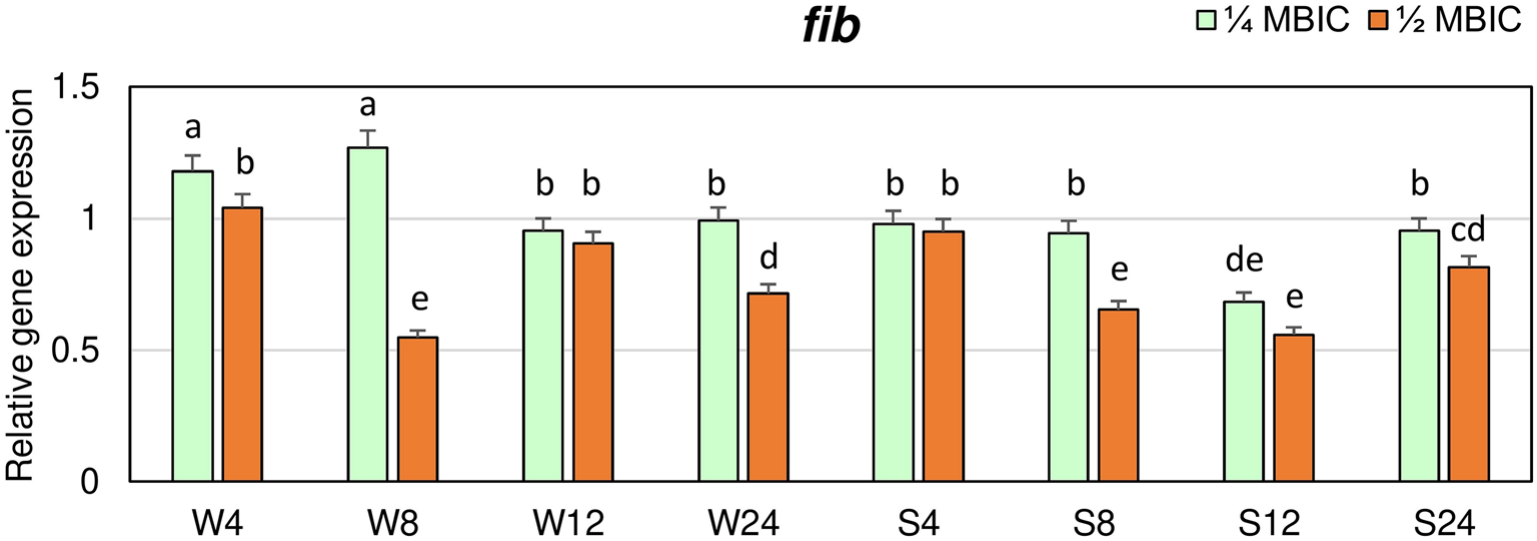
Transcriptional reprogramming of encoding fibrinogen binding protein (*fib*) gene in biofilms of MRSA treated with manuka honey. Gene expression data were normalized to the *rpoB* reference gene. Results are shown as n-fold changes in the target gene expression compared to the control (untreated biofilm). Significant differences in relative gene expression levels among examined MRSA samples were marked by different letters (a, b, d, e, cd, de) (Tukey’s test; *p* < 0.05). W: weak producer of biofilm; S: strong producer of biofilm; 4, 8, 12, 24: time of bacterial growth (h); MBIC: minimum biofilm inhibition concentration. Arithmetic mean and standard deviation, n = 3.

In the strongly adhering strain, expression of *fib* gene at 1/4 MBIC after 4, 8 and 24 h did not significantly differ and was similar as in the untreated biofilm (Fig. 4). A significant decrease of *fib* gene expression in this strain at 1/4 MBIC of manuka honey was observed after 12 h. While, in weakly adhering strain after 4 and 8 h at 1/4 MBIC expression level was 1.2-fold higher in treated biofilm compared to the control. The lowest expression level of *fib* gene at 1/2 MBIC in the weakly adhering strain was observed after 8 h (about twice lower than in the control) and did not significantly differ from expression levels after 8 and 12 h in biofilm of strongly adhering strain.

**Figure 5:**
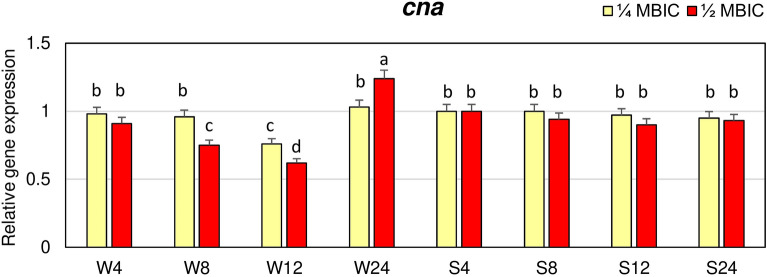
Transcriptional reprogramming of encoding collagen binding protein (*cna*) gene in biofilms of MRSA treated with manuka honey. Gene expression data were normalized to the *rpoB* reference gene. Results are shown as n-fold changes in the target gene expression compared to the control (untreated biofilm). Significant differences in relative gene expression levels among examined MRSA samples were marked by different letters (a, b, c, d) (Tukey’s test; *p* < 0.05). W: weak producer of biofilm; S: strong producer of biofilm; 4, 8, 12, 24: time of bacterial growth (h); MBIC: minimum biofilm inhibition concentration. Arithmetic mean and standard deviation, n = 3.

The expression of *cna* gene in biofilm of strongly adhering strain at 1/4 and 1/2 MBIC of manuka honey did not significantly differ in the subsequent time points and was similar as in the control (Fig. 5). While, in biofilm of weakly adhering strain, significantly lower *cna* expression level was observed after 12 h at 1/4 MBIC and after 8 h and 12 h at 1/2 MBIC compared to the control.

**Figure 6:**
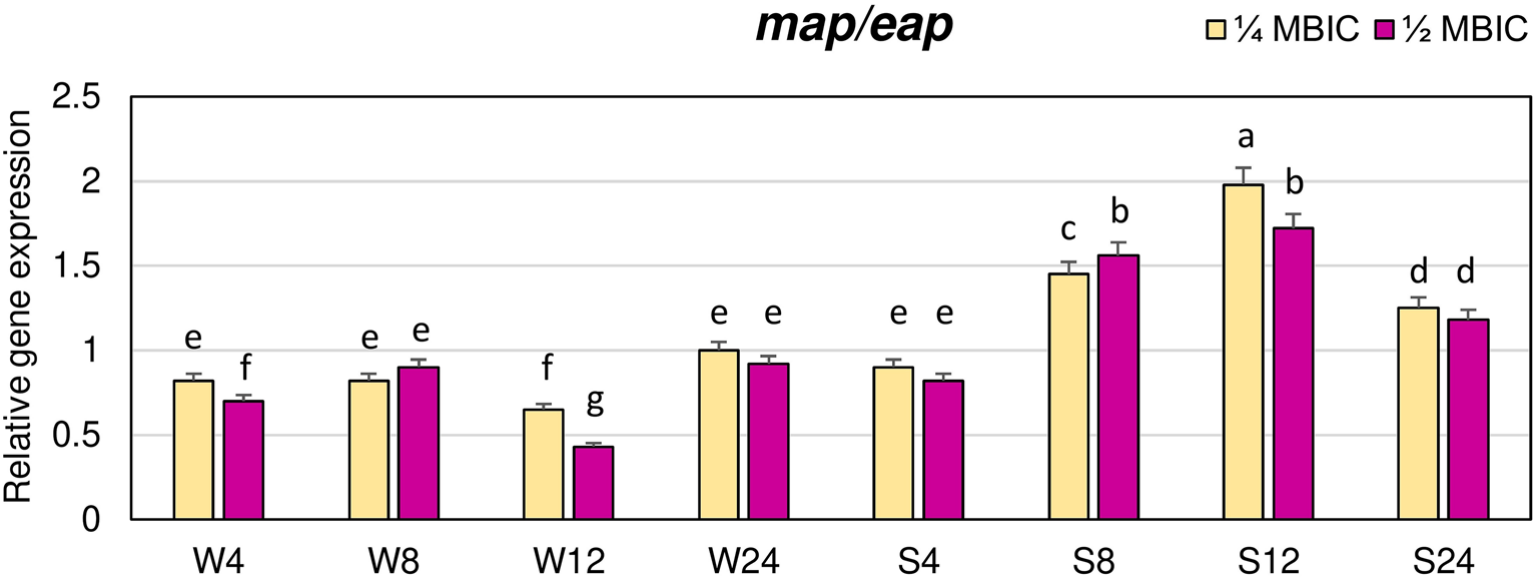
Transcriptional reprogramming of encoding extracellular adherence protein (*map/eap*) gene in biofilms of MRSA treated with manuka honey. Gene expression data were normalized to the *rpoB* reference gene. Results are shown as n-fold changes in the target gene expression compared to the control (untreated biofilm). Significant differences in relative gene expression levels among examined MRSA samples were marked by different letters (a, b, c, d, e, f, g) (Tukey’s test; *p* < 0.05). W: weak producer of biofilm; S: strong producer of biofilm; 4, 8, 12, 24: time of bacterial growth (h); MBIC: minimum biofilm inhibition concentration. Arithmetic mean and standard deviation, n = 3.

The effects of manuka honey on expression of *map/eap* gene encoding an anchorless protein belonging to the group of SERAMs was also investigated. Manuka honey, both at the concentration of 1/4 and 1/2 MBIC, decreased expression level of *map/eap* gene in biofilm of weakly adhering strain after 4, 8 and 12 h of treatment (Fig. 6). The lowest expression level of this gene, equal to 0.4 of the value in the control, was observed after 12 h at 1/2 MBIC. In the strongly adhering strain, the expression level of *map* gene at 1/4 and 1/2 MBIC of manuka honey after 8, 12 and 24 h was higher than in the control.

**Figure 7:**
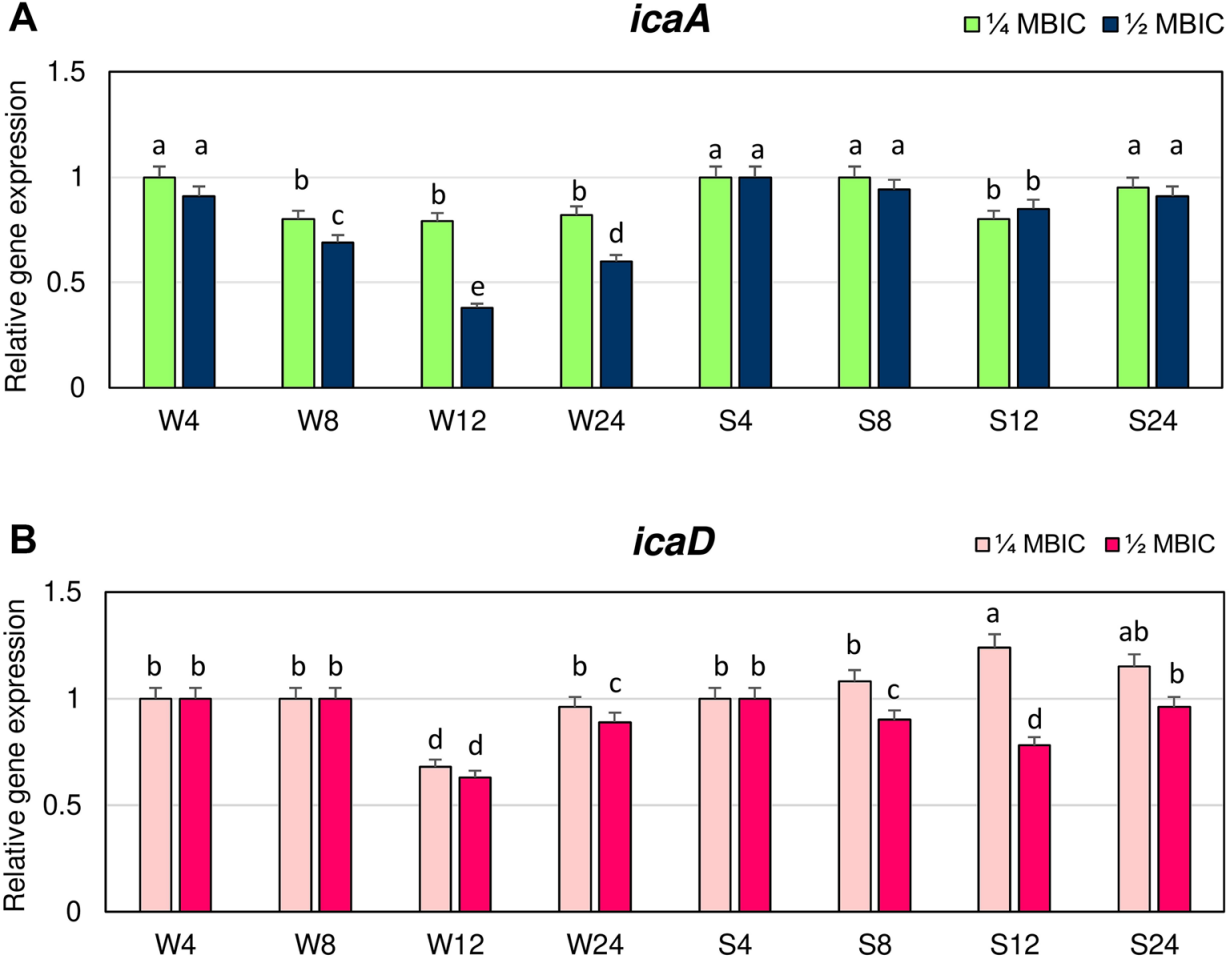
Transcriptional reprogramming of *icaA* (**A**) and *icaD* (**B**) genes in biofilms of MRSA treated with manuka honey. Gene expression data were normalized to the *rpoB* reference gene. Results are shown as n-fold changes in the target gene expression compared to the control (untreated biofilm). Significant differences in relative gene expression among examined MRSA samples were marked by different letters (a, b, c, d, e) (Tukey’s test; *p* < 0.05). W: weak producer of biofilm; S: strong producer of biofilm; 4, 8, 12, 24: time of bacterial growth (h); MBIC: minimum biofilm inhibition concentration. Arithmetic mean and standard deviation, n = 3.

The influence of manuka honey on the expression levels of *icaA* and *icaD* genes from the *ica* operon that are involved in the biosynthesis of glucosamine polymer PIA was also evaluated. Expression levels of the *icaA* gene at 1/4 and 1/2 MBIC of manuka honey in the biofilm of strongly adhering strain after 4, 8 and 24 h did not significantly differ, and they were similar as in untreated biofilm (Fig. 7A).

A significant decrease in expression of *icaA* gene in biofilm of this strain was observed after 12 h. Expression levels of *icaA* in biofilm of weakly adhering strain at 1/4 MBIC were lower after 8, 12 and 24 h than in untreated biofilm and equal to 0.8 of value in the control. Higher concentration of manuka honey (1/2 MBIC) caused a significant reduction in expression levels of *icaA*, that after 12 h (0.4 of expression in the control).

The lowest expression levels of *icaD* in biofilm of the weakly adhering strain were observed after 12 h of treatment with 1/4 and 1/2 MBIC, the values were about 0.7 of the control level (Fig. 7B). While, the lowest expression of *icaD* in biofilm of strongly adhering strain was observed at the same time but only at 1/2 MBIC. Expression level of *icaD* in biofilm of both strains after 12 h at 1/2 MBIC did not significantly differ.

Importantly, the results of factorial analysis of variance (ANOVA) indicated that all tested variables (strain, treatment, growth period) as well as their interactions, significantly affected the expression levels of four target genes (*cna*, *ebps*, *eno*, *fib*) (Table S1) and three target genes (*icaA*, *icaD*, *map/eap*) (Table S2) of MRSA strains. Although, the effect of three interactions (strains × treatment × growth period) on the expression level of the *icaD* gene in both MRSA strains was insignificant (Table S2). Three-factorial ANOVA showed that all variables (strain, treatment, growth period) and their interactions significantly affected bacteria viability in biofilm (Table S3).

## Discussion

MRSA are among the most important biofilm-forming pathogens responsible for hard-to-treat infections including chronic wound infections that are a global health problem. Multi-layered communities of bacteria embedded in the biofilm are difficult to eradicate with antibiotics that were developed primarily to treat acute infections caused by planktonic bacterial populations. Reduction or elimination of established biofilm from infected chronic wounds is of great clinical importance because bacterial biofilms are major contributors to the delay in healing. Damaged tissues of wounds provide a matrix of proteins, including collagen, albumin, fibronectin and fibrinogen, to which pathogens may adhere. Therefore, the search for antibiofilm agents alternative to antibiotics that could be used to treat chronic wounds is necessary. Manuka honey is a promising alternative treatment for wounds covered with staphylococcal biofilm^12,37^. It shows a broad-spectrum antibacterial activity and was reported to be effective against numerous species of bacteria, including MRSA^9,15^.

In our study, we investigated anti-biofilm activity of manuka honey against two MRSA strains of different biofilm-forming abilities. The values of MIC, MBC and MBIC for strongly and weakly adhering strain were the same and equal to 12.5%. Our results are similar to those obtained by Grima et al.^15^, who found that in case of manuka honey containing ≥ 514 mg/kg MGO, MIC values for different MRSA strains were 7% to > 15%. In our study, we used certified manuka honey containing at least 550 mg/kg MGO. Antibacterial activity of manuka honey correlates with the concentration of MGO^38^. This compound showed toxicity to bacterial cells in the wounds by causing bacterial cell lysis. In addition, MGO inhibits flagellation and disrupts bacterial cell division^39–41^. Antibacterial effects of manuka honey are considered to be related also to substantial content of phenolic compounds, flavonoids, and defensins that act synergistically with MGO. The ability of manuka-type honeys to eliminate biofilms of *S. aureus* is due to one or more components present in the honey other than MGO and sugar, such as low pH, hydrogen peroxide, phenolics and other unknown components^12^. Many authors suggest that a multitude of effects caused by manuka honey are induced by more than one of its components^9,15,40^. In our study, we showed that MBIC values of manuka honey measured by viability of biofilms using resazurin^42^ were the same as MIC values. Lu et al.^12^ who investigated manuka honey containing 958 mg/kg MGO, showed that the viability of *S. aureus* cells within the biofilm after treatment with 8% manuka honey was ~ 10% of the untreated control. In our research, we investigated the influence of manuka honey at sub-inhibitory concentrations and we found that metabolic activity of both strong and weak biofilm producer at the presence of manuka honey at 1/2 MBIC in all evaluated time points was significantly lower than in the control, and reduction in metabolic activity of both strains was similar at the same time. The highest reduction of metabolic activity (65–70%, depending on strain and time) compared to the control was observed in both strains after 8 and 12 h. Our results also showed that MRSA metabolic activity in biofilm was significantly inhibited already in the first hours of manuka honey treatment, because after 4 h metabolic activity was reduced about 50% compared to the control. Manuka honey was an effective agent in healing diabetic wounds, significantly reducing wound pH that promoted fibroblast activity, inhibition of protease activity and oxygen release which resulted in reduction of wound size and shortening of healing time^43^. Manuka honey is also thought to stimulate collagen production to repair connective tissue^44^. The observed significant reduction in metabolic activity of bacterial cells in the presence of manuka honey during biofilm formation leads to inhibition of biofilm development and is an additional factor promoting faster wound healing. The antiadhesive properties of manuka honey against *S. aureus* were presented by Maddocks et al.^37^ The activity of manuka honey against formation of staphylococcal biofilm was also demonstrated by other authors^45–47^ but mechanism of its antiadhesive action is not fully understood. Biofilm formation can be subdivided into phases of attachment, accumulation, maturation, and dispersal. Attachment of staphylococcal cells to tissues, including damaged tissues in wound, is mediated by different types of adhesins produced by *S. aureus* that can bind to one or more host extracellular matrix factors^48^. Adhesion of microorganisms to wound bed or other host cells is necessary to initiate infection and biofilm formation, thus interventions that prevent or disrupt biofilms in wounds may improve wound healing outcomes. Looking for the factors alternative to antibiotics, that prevent biofilm formation, we focused on investigation of the effects of manuka honey on the transcriptional profile of genes essential for staphylococcal biofilm formation. We evaluated transcript levels of the genes encoding fibrinogen- (*fib*), collagen- (*cna*), laminin- (*eno*) and elastin binding protein (*ebps*) belonging to the genes encoding MSCRAMMs and *map/eap* gene encoding extracellular adherence protein (Eap) belonging to the group of SERAMs. The influence of manuka honey on the expression of genes associated with MRSA biofilm formation, according to our knowledge, was investigated only by Jenkins et al.^16^, and this study included only the gene coding fibronectin-binding protein. Our research showed that expression levels of genes associated with biofilm formation in the presence of manuka honey at sub-inhibitory concentrations differed from the control, especially in case of weakly adhering strain. The expression of *fib* gene at 1/2 MBIC of manuka honey was lower than in untreated biofilms of both MRSA strains. The highest decrease of *fib* expression was observed after 8 h, and in strongly adhering strain also after 12 h. The amounts of *fib* transcript at this time were about twofold lower compared to untreated biofilms. Adhesion of *S. aureus* to fibrinogen is an important factor of wound infection^21^. Decrease in expression of the MRSA gene coding fibrinogen binding protein after manuka honey treatment probably reduced ability of bacterial cells to adhere to fibrinogen within a wound and thus reduced the probability of the infection onset and biofilm development. Expression of the *eno* and *ebps* genes in biofilms formed by both MRSA strains was also reduced by manuka honey at 1/2 MBIC. However, these genes were most downregulated after 12 h in weak biofilm producing strain. Laminin is an important protein of the blood vessel basement membrane (BM). *eno* gene encodes surface receptors that bind to laminin which enables bacterial cells to cross the BM barrier^18^. Elastin is a polymeric fiber, abundant in lung, skin, and major blood vessels. The EbpS protein is a *S. aureus* receptor that binds to elastin which may facilitate bacterial colonization and pathogenesis^19^. Our results indicate that manuka honey is an effective inhibitor of MRSA adhesion to major components of the extracellular matrix such as laminin and elastin and thus prevents dissemination of bacterial cells and host tissue colonization. Collagen provides the structural support for tissues but in a wounded tissue becomes available to adhering bacterial cells. Adhesion of *S. aureus* to collagen promotes infection and the initiation of biofilm formation^23^. We showed that manuka honey at 1/2 MBIC also reduced the expression level of the *cna* gene, more in biofilm formed by weakly adhering strain. Similar results were obtained for *map/eap* gene. Reduction in expression of this gene in weakly adhering strain was observed in all time points, and the highest decrease occurred after 12 h. While, in the strongly adherent strain, reduction of *map/eap* expression level was observed after 4 h of treatment with 1/2 MBIC of manuka honey, and in subsequent time points the expression level was higher than in control biofilm. Eap, besides the ability to bind to many different glycoproteins of the extracellular matrix, can also form oligomers, and by rebinding to the surface of staphylococcal cells, mediates bacterial agglutination^30^. Maximal production of Eap occurs during the late-exponential phase of growth and is expressed in the stationary growth phase which indicates that this adhesin plays a role in later phase of infection^29^. Manuka honey disrupts regular cell division process of *S. aureus* by inhibiting the activity of murein hydrolase, causing a build-up of septated non-dividing cells, and this mechanism may be described as bacteriostatic activity^49^. Significant increase in the expression level of *map/eap* after 8 h, 12 h and 24 h in strongly adhering strain was probably related to rapid cell division which resulted in attaining the stationary growth phase by this strain earlier compared to the weakly adhering strain. Furthermore, significant increase in *map/eap*, expression may be a defensive response of staphylococcal cells in stationary growth phase to the presence of inhibiting agent by overproduction of Eap oligomers causing agglutination of bacterial cells which probably hinders direct access of antibacterial factor to the cells. Schilcher et al.^50^ suggest that biofilm formation can be induced by conditions that are potentially toxic to bacterial cells, such as the presence of sub-MICs of some antibiotics and that this is strain-specific and related to the induction of stress pathways in *S. aureus* that leads to expression of genes associated with biofilm formation.

Main adhesin responsible for accumulation phase during biofilm formation is the polysaccharide intercellular adhesin (PIA) encoded by *ica* operon, that promotes adhesive interactions among bacterial cells^32^. Extracellular matrix creates a confined environment that provides a physical barrier between a stratified bacterial community and its surrounding environment. The bacterial community is sheltered from the humoral and cell-mediated immune systems and toxic chemicals such as disinfectants and antibiotics^51^. In our study, we investigated influence of manuka honey on the expression of *icaA* and *icaD* genes belonging to *ica* operon. The results of our research showed that in the presence of manuka honey at 1/2 MBIC, the expression of *icaA* and *icaD* genes in biofilm formed by both weakly and strongly adhering strain was the most reduced after 12 h. Manuka honey downregulated more the expression of *icaA* gene in the weakly adhering strain, while in strongly adhering strain 1/4 MBIC caused an increase in the expression level of *icaD*. This may indicate that manuka honey at sub-inhibitory concentration, as many others toxic agents, may induce stress pathways in sessile bacterial population that leads to expression of genes associated with biofilm formation which is the preferred form of bacterial existence.

## Conclusion

Biofilm formation is a significant defense mechanism of bacteria that increases the establishment and duration of infection. The ability of MRSA to form biofilm responsible for hard-to-treat infections make MRSA a serious threat to human health, which is associated with tolerance of staphylococcal biofilms to antibiotics and can lead to failure of antibiotic therapies. For this reason, new therapies should be developed to prevent biofilm formation or actively interfere with the biofilm. The results obtained in this study showed the new mechanism of manuka honey action involved in the preventing of biofilm formation. *S. aureus* cell viability in biofilm and the expression levels of genes involved in the synthesis of binding factors and PIA were reduced at 1/2 MBIC of manuka honey. Manuka honey at sub-inhibitory concentrations caused decrease in the expression levels of *eno*, *ebps*, *fib*, *cna* and *map/eap* genes encoding binding proteins. Down-regulation of these genes may prevent colonization of host tissues including damaged tissues in wounds. Manuka honey also inhibited expression of the genes encoding glucosamine polymer PIA involved in development of multiple layers of sessile bacterial cells protected by a slime substance. Our study showed that the previously unknown mechanism of inhibition by manuka honey of MRSA adhesion to host extracellular matrix factors is the result of reduced expression of crucial genes associated with biofilm formation by MRSA.

## Supplementary information

Supplementary file
