# Supplementary material for: Effect of manuka honey on biofilm-associated genes expression during methicillin-resistant Staphylococcus aureus biofilm formation
Source: Sci Rep. 2020 Aug 11;10:13552. doi: 10.1038/s41598-020-70666-y (PMC7419495; doi:10.1038/s41598-020-70666-y)
Supplement: Supplementary file 1 — Supplementary file [file 41598_2020_70666_MOESM1_ESM.pdf]

Effect of manuka honey on biofilm-associated genes expression during methicillin-resistant Staphylococcus aureus biofilm formation

Barbara Kot*, Hubert Sytykiewicz, Iwona Sprawka, Małgorzata Witeska

Supplementary materials

Table S1 Three-factorial ANOVA analysis of the examined indicators (strains, treatment, growth period) and interactions on relative expression of the tested genes (*cna*, *ebps*, *eno*, *fib*) of MRSA strains

**Indicators**	***cna***	***ebps***	***eno***	***fib***
Strains (S)	$F_{1, 48}$ = 615 (***)	$F_{1, 48}$ = 3,862 (***)	$F_{1, 48}$ = 1,320 (***)	$F_{1, 48}$ = 2,812 (***)
Treatment (T)	$F_{2, 48}$ = 2,040 (***)	$F_{2, 48}$ = 1,250 (***)	$F_{2, 48}$ = 4,505 (***)	$F_{2, 48}$ = 1,565 (***)
Growth period (G)	$F_{3, 48}$ = 1,358 (***)	$F_{3, 48}$ = 812 (***)	$F_{3, 48}$ = 618 (***)	$F_{3, 48}$ = 952 (***)
S × T	$F_{2, 48}$ = 835 (***)	$F_{2, 48}$ = 486 (***)	$F_{2, 48}$ = 435 (**)	$F_{2, 48}$ = 240 (**)
T × G	$F_{6, 48}$ = 425 (**)	$F_{6, 48}$ = 364 (***)	$F_{6, 48}$ = 206 (**)	$F_{6, 48}$ = 137 (*)
S × G	$F_{3, 48}$ = 115 (**)	$F_{3, 48}$ = 208 (**)	$F_{3, 48}$ = 140 (**)	$F_{3, 48}$ = 69 (*)
S × T × G	$F_{6, 48}$ = 62 (*)	$F_{6, 48}$ = 90 (*)	$F_{6, 48}$ = 25 (*)	$F_{6, 48}$ = 44 (*)

* P<0.05; ** P<0.01; *** P<0.001. Tested variables: i) strains – weak and strong biofilm formation; ii) treatment: control, ¼ MIC and ½ MIC; iii) growth period – 4, 8, 12 and 24 h.

Table S2 Three-factorial ANOVA analysis of the examined indicators (strains, treatment, growth period) and interactions on relative expression of the tested genes (*icaA*, *icaD*, *map/eap*) of MRSA strains

**Indicators**	***icaA***	***icaD***	***map/eap***
Strains (S)	$F_{1, 48}$ = 1,450 (***)	$F_{1, 48}$ = 257 (**)	$F_{1, 48}$ = 4,854 (***)
Treatment (T)	$F_{2, 48}$ = 1,220 (***)	$F_{2, 48}$ = 162 (**)	$F_{2, 48}$ = 2,690 (***)
Growth period (G)	$F_{3, 48}$ = 955 (***)	$F_{3, 48}$ = 98 (*)	$F_{3, 48}$ = 2,215 (***)
S × T	$F_{2, 48}$ = 664 (***)	$F_{2, 48}$ = 50 (*)	$F_{2, 48}$ = 1,107 (***)
T × G	$F_{6, 48}$ = 273 (**)	$F_{6, 48}$ = 26 (*)	$F_{6, 48}$ = 814 (***)
S × G	$F_{3, 48}$ = 82 (*)	$F_{3, 48}$ = 12 (*)	$F_{3, 48}$ = 658 (***)
S × T × G	$F_{6, 48}$ = 29 (*)	$F_{6, 48}$ = 0.5 (ns)	$F_{6, 48}$ = 116 (**)

* $P<0.05$; ** $P<0.01$; *** $P<0.001$; ns – non-significant. Tested variables: i) strains – weak and strong biofilm formation; ii) treatment: control, ¼ MIC and ½ MIC; iii) growth period – 4, 8, 12 and 24 h.

Table S3 Three-factorial ANOVA analysis of the studied variables (strains, treatment, growth period) and interactions on the bacteria viability in biofilm

**Indicators**	**Bacteria viability**
Strains (S)	$F_{1, 48}$ = 390 (***)
Treatment (T)	$F_{2, 48}$ = 255 (***)
Growth period (G)	$F_{3, 48}$ = 127 (**)
S × T	$F_{2, 48}$ = 104 (**)
T × G	$F_{6, 48}$ = 38 (*)
S × G	$F_{3, 48}$ = 20 (*)
S × T × G	$F_{6, 48}$ = 14 (*)

* P<0.05; ** P<0.01; *** P<0.001. Tested variables: i) strains – weak and strong biofilm formation; ii) treatment – control, ¼ MIC and ½ MIC; iii) growth period – 4, 8, 12 and 24 h.
